# New Alternative Surgical Technique for Managing Proximal Tibia Chronic Osteomyelitis: Anterior Approach with Establishment of Bone Marrow Communication via Intramedullary Reaming

**DOI:** 10.3390/jcm15010129

**Published:** 2025-12-24

**Authors:** Young-Chang Park, Seung Hyun Kim

**Affiliations:** Department of Orthopaedic Surgery, Severance Hospital, Yonsei University College of Medicine, Seoul 03722, Republic of Korea; yc4508@yuhs.ac

**Keywords:** osteomyelitis, proximal tibia, anterior approach, bone marrow communication, intramedullary reaming

## Abstract

**Background/Objectives**: Surgical treatment of chronic osteomyelitis of the proximal tibia is challenging due to limited soft tissue coverage, poor blood supply, and the weight-bearing function of the bone. Moreover, structural instability following curettage may necessitate fixation with metallic implants, which carries a risk of biofilm formation and often requires multi-stage surgeries. **Methods**: To address these challenges, we developed a new surgical technique combining an anterior approach with establishment of bone marrow communication via intramedullary reaming. The anterior approach provides central access to the infection site, minimizing cortical and cancellous bone loss and eliminating the need for internal fixation. Intramedullary reaming connects the infection site to the systemic circulation, enhancing local blood supply, facilitating intravenous antibiotic delivery, and promoting host immunity. **Results**: Fourteen patients with proximal tibia osteomyelitis were analyzed. The new surgical technique enabled precise targeting of the infection site, substantially reduced unnecessary bone loss, and eliminated the need for internal fixation. Excluding five cases with Cierny–Mader (C-M) classification IV that required fixation due to inherent structural instability, all nine cases with C-M classification III were treated without internal fixation. Two out of three patients with severe post-traumatic osteomyelitis following Gustilo–Anderson type III open fractures were successfully cured. At a mean follow-up of 53.7 months (range: 2.6–104.9 months), 11 of 14 patients were completely cured with a single surgical intervention, corresponding to a 78.6% cure rate. **Conclusions**: This new surgical approach enables one-step surgery, avoids the risks of biofilm formation associated with additional fixation, and enhances treatment efficacy through enhancing host immunity, representing an effective strategy for managing proximal tibia osteomyelitis.

## 1. Introduction

Although combination antibiotic therapy is essential, the cornerstone of curative treatment for osteomyelitis is to surgically eliminate the infection site. However, surgical treatment of proximal tibia osteomyelitis is often challenging, primary due to the anatomical characteristics of the proximal tibia itself. The proximal tibia has limited soft tissue coverage and a relatively poor blood supply, making it more susceptible to infections [1]. Another concern due to the anatomical characteristics of the proximal tibia is weight transmission. The proximal tibia is a direct weight-bearing segment, and the risk of fracture is considerably high due to the loss of cortical and subcortical bone after surgical curettage; therefore, additional fixation using metal implants is often necessary. These metal implants potentiate the risk of bacterial biofilm formation, which compromises the host’s immune system and antibiotic treatment [2] and results in multi-stage surgical treatment [3].

Bone marrow (BM) is a semi-solid tissue that is present in the cancellous bone of the medullary cavity. This tissue has a rich blood flow as it is connected to the systemic circulation through the sinusoid within the medullary cavity [4]. BM serves as a protective niche for hematopoietic and mesenchymal stem cells to maintain the immune system. Establishing a connection between the osteomyelitis lesion and the bone marrow is therefore expected to enhance the penetration and delivery of antibiotics to the infected tissue, while also promoting recruitment of host immune cells, thereby creating a more favorable environment for infection control. Accordingly, intramedullary reaming has been widely applied in chronic osteomyelitis, yielding positive outcomes [5,6,7].

In developing a new surgical technique for proximal tibia osteomyelitis, we focused on two perspectives. First, we designed an anterior approach that minimizes the loss of cortical and subcortical bones of the proximal tibia even after extended curettage and does not compromise the structural integrity of the proximal tibia. The anterior approach has significant advantages in efficient curettage: since the curettage starting point is central, it enables sufficient removal of the lesion without damaging both medial and lateral cortical bones, regardless of whether the lesion is located medially, laterally, or even bilaterally. The tibia comprises a cross-section that is nearly triangular; therefore, even a small defect in the anterior portion will not significantly compromise its structural integrity provided there are no defects in the medial, lateral, and posterior cortices. This factor significantly reduces the need for fixation after surgical removal of the infected area, eliminating the disadvantages caused by biofilm formation on metal implants and requirements for multi-stage surgery. Second, we spatially connected the surgical lesions of the proximal tibia and BM by means of reaming. This technique improves blood flow from BM into the surgical lesions of the proximal tibia, facilitating delivery of intravenous antibiotics and recruitment of host immune cells, thus providing a favorable environment for postoperative antibiotics and host immune responses to treat osteomyelitis.

## 2. Materials and Methods

### 2.1. Patients

We retrospectively reviewed the medical records of 14 patients who had been treated for chronic osteomyelitis in the proximal tibia between May 2016 and August 2025 at Severance Hospital (Seoul, Republic of Korea). This study was approved by the institutional review board (IRB) of Severance Hospital (IRB No. 4-2025-1023). During the study period, all patients who presented to our institution with chronic osteomyelitis of the proximal tibia were treated surgically using this new technique. The present study was conducted retrospectively, and only electronic records were utilized, without any patient-derived materials being accessed. The study protocol was approved by the IRB, which granted a waiver for informed consent.

Information on patient demographics, osteomyelitis characteristics, surgical methods, and treatment outcomes is summarized in Table 1. The male-to-female ratio was 5:2, and the mean age was 57.7 years. The mean duration of symptom onset before surgery was 78.2 months. Ongoing treatment failures for post-traumatic osteomyelitis resulted in exceptionally long symptom durations in patients 4 and 5–23 years (276 months) and 50 years (600 months), respectively. Fortunately, despite these prolonged courses of chronic infection, neither patient showed progression to a Marjolin’s ulcer. For contiguous infections, the symptom duration was calculated from the date of the preceding infected surgery; for post-traumatic infections, it was calculated from the date of the first definite fracture surgery; and for surgical site infections (SSIs), it was calculated from the date of the previous infection-associated surgery. Patient 7 developed an SSI following arthroscopic lateral meniscus repair, and patient 8 developed an SSI after surgery for a giant cell tumor of bone. Osteomyelitis was classified according to the Gustilo–Anderson (G-A) classification, and open fractures were classified according to the Cierny–Mader (C-M) classification (Supplementary Materials S1).

Regarding the infection site, in five cases (patients 3, 7, 9, 10, and 14), the lesion was confined to the metaphysis; in comparison, in eight cases (patients 1, 2, 4, 6, 8, 11, 12, and 13), it had extended to the diaphysis. In one case (patient 5), the lesion was confined to the diaphysis at the level of the tibial tuberosity. The mean size of the infection foci was 13.4 cm^3^. Lesion size was calculated using the ellipsoid formula based on preoperative MRI and CT. In patient 13, there were two separate lesions in the metaphysis and diaphysis, and their combined size was used for the calculation. Patients 3, 8, and 14 had concomitant septic knee arthritis. Five patients were classified as C-M classification IV: four (patients 2, 3, 4, and 6) from post-traumatic osteomyelitis and one (patient 8) from an SSI. The remaining patients were all classified as type III. Regarding host factors, patients 8 and 13 were classified as B^s^: patient 8 had cerebral palsy secondary to traumatic subarachnoid hemorrhage, and patient 13 had type 1 diabetes mellitus. Patient 14 was classified as C due to miliary tuberculosis. Patient 14 eventually died on postoperative day (POD) 14.4 months due to adrenal insufficiency, pleural effusion, ascites and dyspnea caused by exacerbation of miliary pulmonary tuberculosis, although the osteomyelitis was resolved. The most common pathogen was *Staphylococcus*: there were two cases (patients 9 and 11) of methicillin-sensitive *Staphylococcus aureus* (MSSA), two cases (patients 1 and 4) of coagulase-negative *Staphylococcus* (CoNS), and two cases (patient 6 and 8) of methicillin-resistant *Staphylococcus aureus* (MRSA). Patient 3 presented with a fungal infection caused by *Candida albicans* (*C. albicans*); in comparison, patients 13 and 14 had tuberculosis (TB) infections. Pathogens could not be detected in three cases (patients 7, 10, and 12).

### 2.2. Surgical Technique

**Figure 1:**
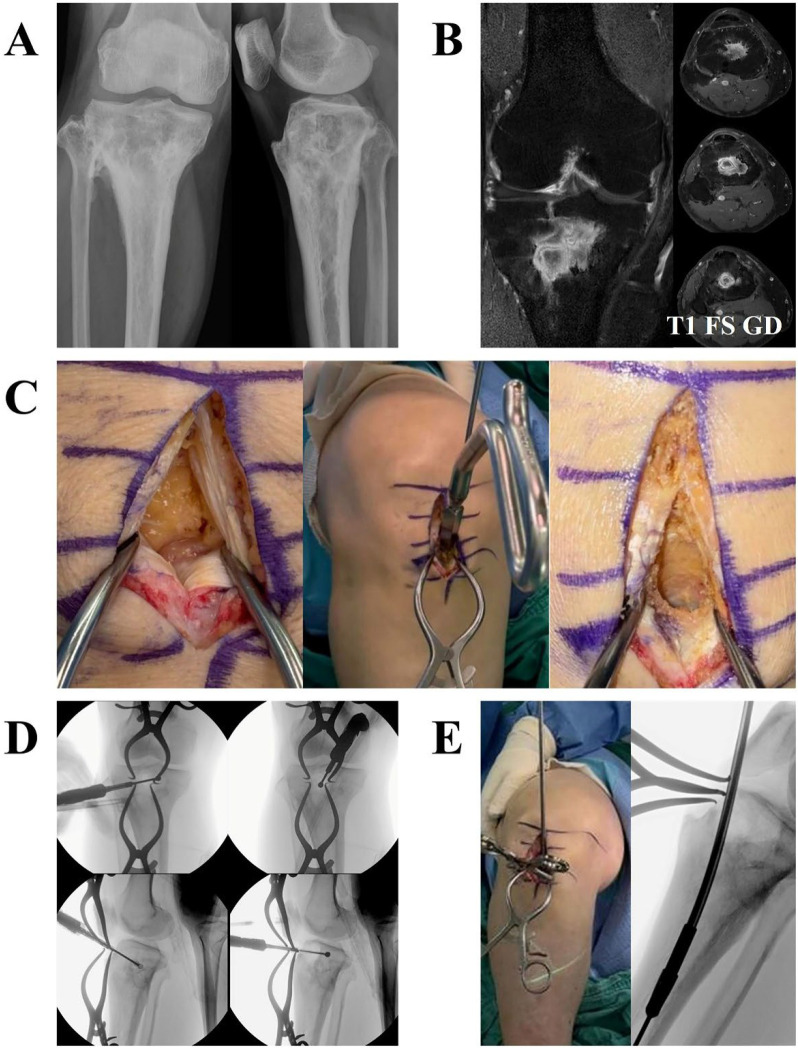
Illustration of the new surgical technique (anterior approach and osteoimmune activation). This figure shows, in sequence, the imaging findings and the surgical procedure of patient 9. (**A**) X-ray at diagnosis. (**B**) MRI at diagnosis. The infection site was located at the center of the metaphysis. Therefore, regardless of whether the approach was made medially or laterally, considerable unnecessary bone defects to access the infection site requiring fixation with a metal implant were expected to remain after curettage of the lesion. (**C**) Anterior approach. In this case, an anterior approach was performed through the patellar tendon (transpatellar approach). The patellar tendon was split longitudinally, and the infrapatellar fat pad was removed (**left** panel). An opening in the proximal tibia was then created using an awl and a proximal reamer, instruments used for tibia nailing (**middle** panel). As a result, a bone window large enough to allow access for a curette and burr was obtained (**right** panel). (**D**) C-arm fluoroscopic images demonstrating extensive intramedullary curettage using a burr. Through the window, curettage can be freely performed in any direction and depth without causing any damage to the cortical bone. (**E**) To increase blood flow and trigger osteoimmune activity, communication occurs between the infected site and the bone marrow. Similarly, communication can be easily established using tibia nailing instruments such as a ball tip guide and a flexible reamer. Abbreviation: T1 FS GD, T1-weighted fat-suppressed gadolinium-enhanced.

The new surgical technique is illustrated in Figure 1, which shows intraoperative photographs from patient 9. The infection site was located at the center of the metaphysis (Figure 1B). Therefore, regardless of whether a medial or lateral approach is used, creating a large bone window to access the lesion inevitably results in substantial and unnecessary bone loss. Such large bone loss compromises the structural stability of the proximal tibia, ultimately necessitating supplementary fixation with metallic implants. On the other hand, by using the anterior approach, these problems can be easily overcome. A sufficiently large bone window required for lesion access is created above the tibial tuberosity through a trans-patellar approach (Figure 1C). In this case, the area around the tibial tuberosity is not involved in weight transmission, and the medial, lateral, and posterior cortices—which bear weight—remain completely intact, eliminating the need for supplementary fixation. Another advantage of the anterior approach is that it facilitates successful curettage. Because the bone window to the lesion is located at the center of the lesion, curettage can be freely performed in any direction and depth (Figure 1D). Conversely, if the bone window is positioned at either the medial or lateral edge of the lesion, surgical access is severely restricted. Osteoimmune activation can be achieved using a very simple method. A flexible reamer is used to establish a physical connection between the medullary canal and the lesion, increasing blood flow to the lesion and osteoclast recruitment, thereby activating osteoimmune responses (Figure 1E). These procedures can be readily carried out without the need for specialized equipment. The bone window was prepared using the awl and proximal drill of the tibia nail system: an Expert Tibia Nail (ETN) (DePuy Synthes, Oberdorf, Switzerland) (Figure 1C); in comparison, a flexible reamer from the same system: an Expert Tibia Nail (ETN) (DePuy Synthes, Oberdorf, Switzerland) was utilized to create a physical connection between the medullary canal and the lesion (Figure 1E). For six patients (patients 5, 9, 10, 11, and 12), the surgeries were conducted exclusively using this technique.

Using the aforementioned surgical technique as a foundation—anterior approach and osteoimmune activation—additional or modified procedures were performed as required according to each case. Because there was a total of three cases in which septic arthritis of the knee occurred concurrently with osteomyelitis, synovectomy was employed as an additional procedure. Arthroscopic debridement was additionally and independently performed in patient 14, combined with the new surgical technique. Open synovectomy via an anterior approach was performed in patients 3 and 8, which resulted in structural instability. Together with the two aforementioned cases, a total of five C–M classification IV cases, including three infected nonunion cases (patients 2, 4, and 6), were identified; all required additional intramedullary nail fixation. Insertion of an Expert Tibia Nail (ETN) (DePuy Synthes, Oberdorf, Switzerland) was additionally performed in all infected nonunion cases. Following vigorous anterior open synovectomy, patient 3 developed a ‘blow-knee’ state, for which a temporary arthrodesis was performed using a Küntscher nail (now discontinued) and bone cement. In patient 8, a massive bone defect occurred; reconstruction was achieved by insertion of an Ender’s nail (Smith and Nephew, Watford, England) in combination with bone cement. In the case of patient 7, additional curettage was performed at the satellite infection site on the diaphysis. In patient 13, an anterior approach via the lateral aspect of the patellar tendon was attempted. In patients 1 and 2, removal of the existing metallic implants was additionally performed. The above details will be further specified in Section 3, accompanied by figures.

### 2.3. Postoperative Antibiotics

The preoperative blood test results associated with infection for each patient, combined with their postoperative antibiotic treatment histories, are summarized in Table 2. Leukocytosis was observed in two cases, erythrocyte sedimentation rate (ESR) in eleven cases, and C-reactive protein (CRP) in nine cases.

Because there are currently no clear guidelines regarding antibiotic use [8], antibiotic therapy was primarily guided by the Prosthetic Joint Infection: Clinical Practice Guidelines of the Infectious Diseases Society of America [9]. Beyond culture-based guidance, when empirical coverage for Gram-negative organisms was considered clinically necessary, intravenous aminoglycosides and oral ciprofloxacin were administered as part of combination therapy. Intravenous (IV) antibiotics were administered for a mean duration of 12.3 days (range: 2–28 days), and oral antibiotics were given for a mean duration of 18.5 days (range: 7–40 days). The total duration of antibiotic therapy was a mean of 29.2 days (range: 7–58 days). Antituberculosis drugs were administered orally for six months based on the regimens listed in Table 2.

### 2.4. Assessment of Treatment Outcomes

Treatment outcomes were clinically accessed. Complete resolution was defined as a condition in which no evidence of infection was identified on physical examination, imaging studies (X-ray, CT, MRI, or whole-body bone scan), or laboratory evaluations (ESR and CRP). Unfortunately, parameters such as functional scores and gait status were not included in the assessment.

Consequently, complete resolution was achieved in 11 of the 14 patients. Among the three patients who experienced treatment failure (Patients 3, 6, and 8), only Patient 3 demonstrated findings consistent with type IIIA osteomyelitis and subsequently underwent reoperation. The two remaining patients with type IIA osteomyelitis were treated with skillful neglect instead of reoperation: Patient 8 had a systemic history of cerebral palsy resulting from traumatic subarachnoid hemorrhage, and patient 6 declined further surgical intervention after experiencing fatigue from prolonged vacuum-assisted granulation therapy and repeated skin grafts performed for a large traumatic degloving injury. For Patient 3, *C. albicans* detected on culture was initially considered a contamination-related false-positive result; however, it was later confirmed to represent a true fungal infection, and appropriate antifungal therapy was not administered. In the other two patients, MSSA was initially isolated, but the final culture results identified MRSA, which led to suboptimal antibiotic therapy.

## 3. Results

### 3.1. The New Surgical Technique Enables Precise Targeting of the Infection Site Minimizing Unnecessary Bone Loss

**Figure 2:**
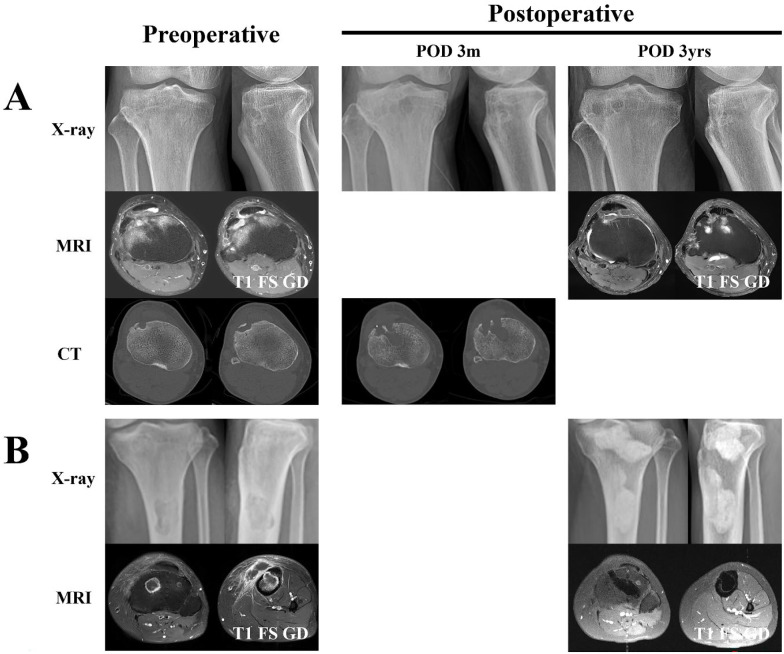
The anterior approach enables precise targeting of the infection site and preserves the surrounding bone. (**A**) surgical outcome of patient 7. An anterior approach was achieved via the lateral aspect of the patellar tendon, representing a slightly modified anterior approach compared to that shown in Figure 1. Postoperative CT at 3 months demonstrates adequate targeting without unnecessary bone loss. Bone cement filling or fixation with metallic implants was not required. MRI at 3 years postoperatively shows regeneration of the subcortical bone. (**B**) Surgical outcome of patient 13. A trans-patellar approach, as shown in Figure 1C, was performed for the metaphyseal infection site, and a direct approach through the diaphyseal abscess was additionally undertaken. Bone cement filling was performed due to two separate infection foci in the metaphysis and diaphysis, even though fixation was not needed. The position of the bone cement on MRI three years after surgery demonstrates that the infection foci were accurately targeted during curettage. Abbreviations: POD, postoperative day; m, months; yr, years; T1 FS GD, T1-weighted fat-suppressed gadolinium-enhanced.

The results presented in Figure 1D demonstrate that the new surgical technique facilitates accurate targeting of metaphyseal lesions. Figure 2 provides illustrative examples supporting these findings. Figure 2A shows the treatment outcome of a 63-year-old male patient (Patient 7) who developed an SSI following arthroscopic lateral meniscus repair. CT performed three months postoperatively demonstrated that the bone defect resulting from the curettage corresponds to the lesion, indicating accurate lesion targeting without unnecessary bone loss. MRI performed three years after surgery showed regeneration of the subcortical bone. Figure 2B shows the treatment outcome of a 33-year-old female patient (Patient 13) with TB bone infection presenting with separate infection foci in the metaphysis and diaphysis. A trans-patellar approach, as shown in Figure 1C, was performed for the metaphyseal infection site, and a direct approach through diaphyseal abscess was additionally undertaken. The position of the bone cement on MRI three years after surgery demonstrates that the infection foci were accurately targeted. Bone cement filling was performed due to two separate infection foci in the metaphysis and diaphysis, even though fixation was not required.

### 3.2. The New Surgical Technique Largely Eliminates the Need for Internal Fixation with Metallic Implants After Curettage

**Figure 3:**
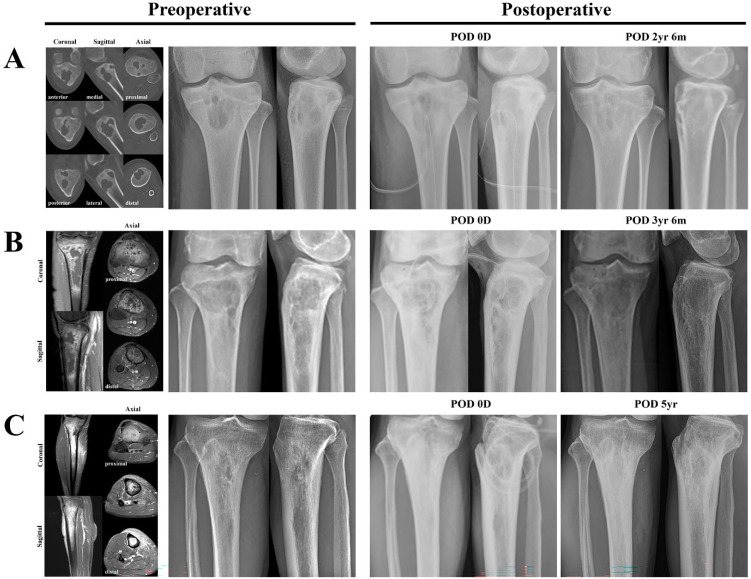
The anterior approach enables the cortical bone to be preserved intact, thereby largely eliminating the need for fixation with metallic implants in osteomyelitis cases below C-M classification. All cases, (**A**–**C**) underwent surgery via the trans-patellar approach, as illustrated in Figure 1. (**A**) Surgical outcome of patient 10. On preoperative CT, extensive multifocal osteolytic lesions are observed in the metaphysis. However, despite extensive trabecular bone destruction, the integrity of the cortical bone is relatively well preserved. The anterior approach causes no cortical bone damage, excluding the bone opening around the tibial tuberosity; thus, even after extensive curettage, the structural integrity of the tibia is preserved, allowing fixation to be avoided. Surgical outcome of patient 11 (**B**) and patient 12 (**C**). (**B**,**C**) are cases in which the infection site has extended longitudinally within the medullary cavity from the metaphysis to the diaphysis. In these cases, in addition to metaphyseal burring, the infection foci in the diaphysis were removed using a large-diameter reamer during medullary reaming (Figure 1E), which was performed to create communication with the bone marrow. Although the areas of curettage were greatly expanded, fixation with metallic implants was still not required. Abbreviations: POD, postoperative day; D, days; m, months; yr, years; T1 FS GD, T1-weighted fat-suppressed gadolinium-enhanced; C-M, Cierny–Mader.

Another advantage of this new surgical technique is that it enables precise curettage of the infectious site while avoiding unnecessary removal of the surrounding cancellous bone and, in particular, preventing cortical bone damage, thereby eliminating the need for metallic implants for internal fixation. This approach, in turn, may prevent biofilm formation on implants and provide benefits in the treatment of osteomyelitis. As a result, internal fixation was required in all five patients with infected nonunion because of their inherent structural instability; in comparison, none of the nine patients with C-M classification III lesions required additional fixation to compensate for bone loss after curettage. This indicates that the need for fixation arose not from the new surgical technique itself, but from the failure of the prior fracture treatment. Figure 3 provides illustrative examples supporting above findings. All cases, A, B, and C, were treated exclusively with the new surgical technique presented in Figure 1. Figure 3A (Patient 10, a 22-year-old female) shows an extensive osteolytic cancellous bone infection occupying more than one-fourth of the metaphysis. If surgery had been performed using the conventional medial approach (as the lesion was located closer to the medial cortex and thus considered more favorable for medial than lateral access), curettage would have caused extensive bone loss in the medial cortex and cancellous bone, necessitating additional internal fixation with metallic implants and bone cement filling. However, by using the anterior approach, the curette and burr can directly access the lesion, minimizing the loss of surrounding cancellous bone and preventing damage to both the medial and lateral cortices, thereby eliminating the need for internal fixation or bone cement filling. As a result, the proximal tibia maintained structural stability during the 2.5-year follow-up period without the need for additional fixation, with complete resolution of osteomyelitis.

Figure 3B (patient 11, a 76-year-old female) and Figure 3C (patient 12, a 71-year-old male) illustrates examples of extensive osteomyelitis extending from the metaphysis to the diaphysis. Both cases were treated using the new surgical technique and remained fracture-free without additional fixation or bone cement filling for 3.5 and 5 years, respectively, with complete resolution of osteomyelitis.

### 3.3. Outcomes of the New Surgical Technique for the Treatment of Severe Post-Traumatic Chronic Osteomyelitis

**Figure 4:**
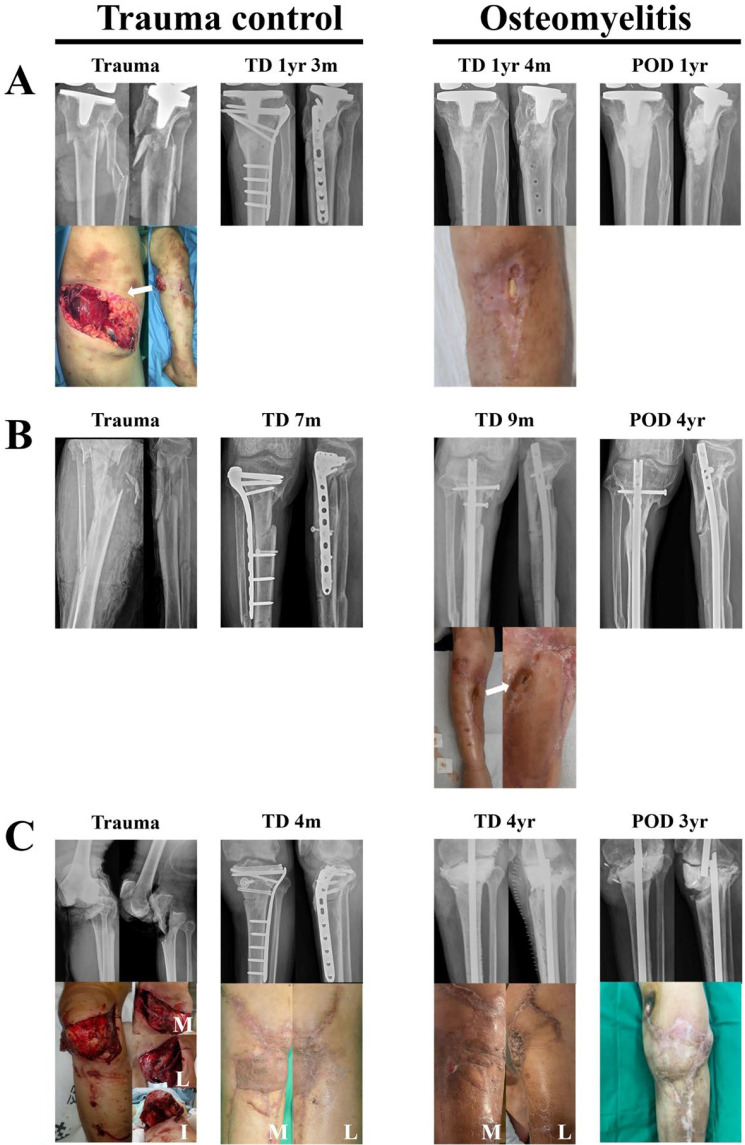
Outcomes of the new surgical technique for the treatment of severe post-traumatic osteomyelitis. Severe open fractures frequently result in osteomyelitis that is challenging to manage. (**A**) Patient 1: The patient had a G-A classification II open fracture in the setting of a previous joint replacement. Although bone union was achieved after surgery, C-M classification IIIA osteomyelitis developed at POD 1 year 4 months, and the infection was not resolved even after metal implant removal. Curettage was performed via an anterior approach through the open wound and tibial tuberosity, and communication with the marrow was established. The bone defect was then filled with vancomycin-loaded cement, resulting in the resolution of osteomyelitis. No additional fixation was required. (**B**) Patient 2: Infected nonunion developed following a G-A classification IIIB open fracture, C-M classification IVA. The preexisting plate and screws were removed, and a tibia intramedullary nail was inserted while applying the new surgical technique, resulting in the resolution of osteomyelitis. The arrow indicates an open wound. (**C**) Patient 3: Bone union was achieved following a severe G-A classification IIIB open fracture with knee dislocation; however, the persistently recurring low-grade infection (below C-M classification IIA) worsened, resulting in active infection (C-M classification IVB^L^) accompanied by septic knee arthritis. Via an anterior approach, all implants were removed, and thorough resection of infected bone and synovectomy were performed. To resolve the resulting ‘blow-knee’ condition, temporary arthrodesis was performed using a Küntscher nail and vancomycin-loaded cement, with communication established with the marrow; however, the treatment was not successful. Abbreviations: TD, trauma date; POD, postoperative day; yr, years; m, months; M, medial; L, lateral; I, inferior; G-A, Gustilo–Anderson; C-M, Cierny–Mader.

G-A classification III severe open fractures frequently lead to residual osteomyelitis even after bony union. In this study, we report three cases (patients 2, 3, and 6) of osteomyelitis resulting from G-A classification III open fractures. Figure 4 illustrates cases of post-traumatic osteomyelitis treated using the new surgical technique. Figure 4A (patient 1, a 69-year-old male) shows an open periprosthetic fracture classified as G-A type II. Bone union was achieved; however, infection occurred, leading to the removal of the internal fixation. Despite this, osteomyelitis persisted, and at trauma date (TD) 1 year and 4 months, the patient continued to exhibit C-M type IIIA osteomyelitis. With a modification of the new surgical technique, the anterior approach was achieved through the wound opening and the bone defect was filled with vancomycin-loaded cement (45 mg/g of cement) after curettage (Table 2). Figure 4B (patient 2, a 66-year-old male) illustrates a case of infected nonunion following an open fracture classified as G-A type IIIB. At TD 9 months, the patient exhibited infected nonunion accompanied by C-M classification IVA osteomyelitis. Osteomyelitis was treated using the new surgical technique, with removal of the previous fixation devices and insertion of a new intramedullary nail additionally. Figure 4C (patient 3, a 53-year-old male) also illustrates a case of osteomyelitis following an open fracture classified as G-A type IIIB, combined with knee joint dislocation. A low-grade infection (C-M classification below IIA) developed and repeatedly showed periods of improvement, but eventually worsened, and at TD 4 years, the patient developed an active infection (C-M classification IVB^L^) accompanied by septic knee arthritis. Through an anterior approach and open synovectomy, all implants were removed, and extensive resection of infected bone and synovectomy were performed, resulting in a ‘blow-knee’ condition. Temporary arthrodesis was added using a Küntscher nail and vancomycin-loaded cement (45 mg/g of cement). While the cases illustrated in Figure 4A,B were successfully treated for osteomyelitis, the case shown in Figure 4C was unsuccessful.

The new technique also demonstrated efficacy in severe post-traumatic osteomyelitis following Gustilo–Anderson type III fractures. Two out of three such cases were successfully treated, highlighting the potential utility of this approach even in high-risk patients with complex infection patterns.

### 3.4. Long-Term Follow-Up Results of the Anterior Approach and Osteoimmune Activation

The clinical outcomes of all 14 cases are summarized in Table 1. The mean follow-up period after osteomyelitis surgery was 53.7 months. Eleven cases were successfully treated, resulting in a final osteomyelitis resolution rate of 78.6% using the new surgical technique. Although there are no established guidelines for antibiotic therapy in osteomyelitis, antibiotic selection was managed under the supervision of the hospital’s infectious disease department, and the duration of administration, averaging 4.2 weeks (range, 1.0–8.3 weeks), was based on conventional consensus. Since antibiotic use did not differ significantly from consensus treatment, the observed 78.6% resolution rate is considered to be primarily attributable to the effect of the new surgical technique. Excluding five C-M classification IV cases, in which fixation was unavoidable due to inherent structural instability, internal fixation was not required in any of the nine C-M classification III cases. All patients were treated with a single surgical intervention.

**Figure 5:**
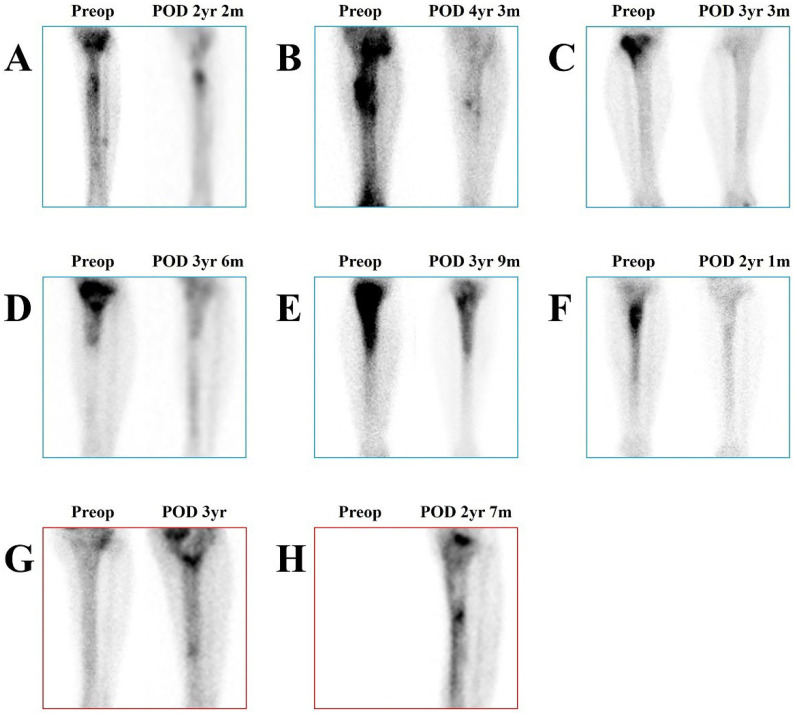
Long-term follow up results of the anterior approach and osteoimmune activation. Tc-99m MDP uptake on WBBS was interpreted as a measure of osteomyelitis activity. (**A**) Patient 2; (**B**) patient 4; (**C**) patient 7; (**D**) patient 11; (**E**) patient 12; (**F**) patient 13; (**G**) patient 3; and (**H**) patient 6. A preoperative WBBS was not performed for case H. The resolved group (**A**–**G**) is outlined in blue, and the treatment-failure group (**G**,**H**) is outlined in red. In the resolved group, osteomyelitis activity either resolved or markedly decreased, indicating that the new surgical technique, combining the anterior approach and osteoimmune activation, is highly effective. Exceptionally, in case (**E**), although considerable Tc-99m MDP uptake remained, there were no clinical symptoms of osteomyelitis. In contrast, cases (**G**,**H**) showed residual osteomyelitis consistent with the Tc-99m MDP uptake, corresponding to C–M classification IIIA and IIA, respectively, and ultimately resulted in treatment failure. Abbreviations: Tc-99m MDP, Technetium-99m methylene diphosphate; WBBS, whole-body bone scintigraphy; Preop, preoperative; POD, postoperative day; m, months; yr, years; C-M, Cierny–Mader.

There has been considerable debate regarding whether Tc-99m MDP (Technetium-99m methylene diphosphate) uptake on whole-body bone scintigraphy (WBBS) accurately reflects the activity of osteomyelitis [10,11] and several adjunctive imaging strategies have been proposed to enhance diagnostic performance [12,13]. However, the fundamental limitation of Tc-99m MDP uptake in assessing osteomyelitis activity remains its high sensitivity but poor specificity. which leads to a substantial rate of false-positive findings. Although its low specificity can lead to false-positive diagnoses during the initial evaluation, the high sensitivity of this modality becomes advantageous during postoperative follow-up, as it allows for reliable detection of potential recurrence. Therefore, long-term follow-up outcomes of the new surgical technique were assessed by means of WBBS, since the increased uptake on a WBBS can be considered as an indicator of osteomyelitis activity (Figure 5). In the resolved group (A–G), osteomyelitis activity either resolved or markedly decreased, indicating that the new surgical technique, combining the anterior approach and osteoimmune activation, is highly effective.

## 4. Discussion

Chronic osteomyelitis of the proximal tibia remains a significant surgical challenge due to the bone’s limited soft tissue coverage, poor vascularity, and weight-bearing nature. Traditional surgical management often necessitates multi-stage procedures with metallic internal fixation to maintain structural integrity after extensive curettage. These implants, however, increase the risk of bacterial biofilm formation, compromise the host immune response, and may contribute to treatment failure [2,3]. Consequently, there is a critical need for surgical strategies that preserve bone structure, minimize implant use, and enhance host immunity.

Various surgical techniques have been reported to improve the clinical outcomes of patients with proximal tibia osteomyelitis. These surgical techniques generally consist of three main stages: removing the infected area, managing the dead space caused by the bone defect through surgical removal, and fixation using metal implants, which leads to multi-step surgeries. Recently reported new surgical techniques are mainly related to the management of dead space and the regeneration of bone defects. Traditionally, antibiotic-loaded cements [14] have been widely used for dead space management; however, antibiotic-loaded calcium sulfate [15] or antibiotic-impregnated biodegradable carriers [16] are emerging as new alternatives. Moreover, regarding bone regeneration, bone graft represented the gold standard in the past; more recently, however, various induced membrane techniques [17,18,19] and bone transport techniques [20] have been reported to produce satisfactory results. While these new surgical techniques have improved the outcomes of patients with proximal tibia osteomyelitis, unfortunately, additional fixation using metal implants is still required, necessitating multi-stage surgery to overcome bacterial biofilm formation and remove the implants. At present, however, the only method established to avoid fixation is temporary external fixation [20,21], which also requires multi-stage surgery. In this study, the anterior approach enables direct and central access to the infection site, minimizing unnecessary cortical and cancellous bone loss. This technique preserves structural integrity and largely eliminates the need for internal fixation in patients with osteomyelitis classified as C-M III. Indeed, in our cohort, none of the nine patients with C-M type III lesions required metallic implants, whereas fixation was only necessary in five patients with C-M type IV lesions due to inherent structural instability. This finding demonstrates that the anterior approach not only facilitates precise lesion targeting but also reduces the risks associated with metallic implants, including biofilm formation and the need for multi-stage surgeries.

Intramedullary reaming serves a dual purpose: it establishes a connection between the systemic circulation and the infection site, thereby improving local blood supply and enhancing antibiotic delivery, and it activates osteoimmune responses. By promoting osteoclast recruitment and facilitating immune-mediated bone defense, this technique supports both infection clearance and bone regeneration. At present, osteoimmunology, the study of the interaction between the immune system and bone metabolism, has emerged as a new field and is undergoing development [22,23,24,25]. Macrophages, derived from monocyte lineage in the BM, play a key role in innate immunity to defend the host against various pathogens. Macrophages are polarized into M1 and M2 macrophages. The formers macrophages are essential for eliminating pathogens and promoting inflammation, producing pro-inflammatory cytokines such as IL-6 and TNF-α; in comparison, the latter macrophages are crucial for promoting tissue repair and resolving inflammation, producing anti-inflammatory cytokines such as IL-10 and TGF-β [26]. It is widely recognized that osteoclasts, macrophages of the musculoskeletal system, drive bone remodeling through the receptor activator of the NF-κB ligand (RANKL) signaling pathway [27]. In addition to bone remodeling, osteoclasts play an important role in immunity against bone infection [28]. *Staphylococcus aureus* (*S. aures*), the most common pathogen for osteomyelitis, induces osteoclastogenesis through the NF-κB signaling pathway [29] and even directly infects osteoclasts and replicates within them [30]. The fact that a single surgical intervention alone achieved a high success rate of 78.6% (11 out of 14 patients) raises the possibility that enhanced osteo-immune activation played a major role.

This study includes several limitations: the retrospective design; the very small sample size; the lack of a control or comparison group treated with conventional techniques; and the heterogeneity of clinical presentations, pathogens, and host factors. Issues such as the absence of functional or biomechanical assessments, potential selection bias, and the difficulty in isolating the effect of the surgical procedure from antibiotic treatment or natural disease progression may also be open to debate. Additionally, long-term functional outcomes, pain, and biomechanical assessments were not systematically evaluated. Nevertheless, the mean follow-up of 53.7 months and the consistent resolution of osteomyelitis in the majority of cases suggest that this technique provides durable infection control. Future studies with larger cohorts, comparative designs, and functional outcome assessments are warranted to validate and further refine this approach.

## 5. Conclusions

The anterior approach combined with osteoimmune activation via intramedullary reaming is an effective and reliable surgical strategy for chronic osteomyelitis of the proximal tibia. The anterior approach enables precise targeting of the infection site, minimizes unnecessary cortical and cancellous bone loss, preserves structural integrity, and largely eliminates the need for internal fixation in C-M classification III lesions, reducing the risk of biofilm formation. Osteoimmune activation via intramedullary reaming connects the infection site to the systemic circulation, enhancing local blood supply, facilitating intravenous antibiotic delivery, and promoting osteoclast-mediated bone defense. In this study, a new surgical technique of single-stage procedure resulted in complete resolution in 11 out of 14 patients. This technique offers a promising alternative to conventional multi-stage surgeries and improves overall treatment outcomes for proximal tibia chronic osteomyelitis.

## Figures and Tables

**Table 1:** Summary of patient demographics, infection condition, surgical methods and treatment outcomes.

		Patient No.	Sex	Age	Sx Duration (mo)	Location	Size ^1^ (cm^3^)	Combined Septic Knee	Cierny–Mader Class	Gustilo–Anderson Class	Pathogen	I/F Using Implants	Bone Cement Filling	F/U Period (mo)	Treatment Outcome
Contiguous	Post-traumatic	1	M	69	16.1	Meta to Dia	13.4	-	IIIA	II	CoNS	-	yes	12.0	resolved
		2	M	66	7.6	Meta to Dia	14.3	-	IVA	IIIB	GBS	yes	-	50.5	resolved
		3	M	56	49.6	Meta	121.8	yes	IVB ^L^	IIIB	*C. albicans*	yes	-	87.4	IIIA ^2^
		4	M	57	276.0	Meta to Dia	82.9	-	IVA	NA	CoNS	yes	-	75.4	resolved
		5	M	71	600.0	Dia	18.5	-	IIIA	NA	*S. marcescens*	-	-	46.5	resolved
		6	F	57	1.9	Meta to Dia	17.8	-	IVB ^L^	IIIB	MRSA	yes	-	104.8	IIA ^2^
	SSI	7	M	63	35.9	Meta	10.6	-	IIIA	-	negative ^3^	-	-	39.2	resolved
		8	M	43	12.3	Meta to Dia	104.7	yes	IVB ^S4^	-	MRSA	yes	yes	61.3	IIA ^2^
Idiopathic		9	M	54	3.0	Meta	13.1	-	IIIA	-	MSSA	-	-	2.6	resolved
		10	F	23	12.0	Meta	10.2	-	IIIA	-	negative ^3^	-	-	28.7	resolved
		11	F	76	4.0	Meta to Dia	23.5	-	IIIA	-	MSSA	-	-	63.3	resolved
		12	M	71	12.0	Meta to Dia	38.2	-	IIIA	-	negative ^3^	-	-	60.5	resolved
MTB		13	F	33	60.0	Meta to Dia	11.9	-	IIIB ^S5^	-	MTB	-	yes	104.9	resolved
		14	M	69	5.0	Meta	14.0	yes ^7^	IIIC ^6^	-	MTB	-	yes	14.4	resolved ^8^
Mean ± SD (min-max)		-	-	57.7 ± 15.4 (23–76)	78.2 ± 166.1 (1.90–600.0)	-	13.4 ± 38.2 (10.2–121.8)	-	-	-	-	-	-	53.7 ± 32.6 (2.6–104.9)	-

^L^ local factor, ^S^ systemic factor, ^1^ Size of the infected area, calculated using the ellipsoid formula based on preoperative MRI or CT scans; ^2^ assessed using the Cierny–Mader classification; ^3^ no growth on culture; ^S4^ CP secondary to traumatic SAH; ^S5^ T1DM; ^6^ miliary tuberculosis; ^7^ arthroscopic debridement with concurrent procedure; ^8^ although the osteomyelitis was cured, the patient eventually died on POD 14.4 months due to adrenal insufficiency, pleural effusion, ascites and dyspnea caused by exacerbation of miliary pulmonary tuberculosis. Abbreviations: No, number; Sx, symptom; mo, months; Class, classification; I/F, internal fixation; F/U, follow-up; SSI, surgical site infection; MTB, *Mycobacterium tuberculosis*; Meta, metaphysis; Dia, diaphysis; CoNS, coagulase-negative *Staphylococcus*; GBS, *Streptococcus*, beta-hemolytic, Group B; *C. albicans*, *Candida albicans*; *S. marcescens*, *Serratia marcescens*; MRSA, methicillin-resistant *Staphylococcus aures*; MSSA, methicillin-sensitive *Staphylococcus aures*; CP, cerebral palsy; SAH, subarachnoid hemorrhage; T1DM, type 1 diabetes mellitus; SD, standard deviation.

**Table 2:** Summary of initial infection blood test results and antibiotics administration history.

Patient No.	Laboratory Results at Diagnosis			Medical Treatment (Antibiotics)							
				IV			PO			Anti-Loaded Cement	
	WBC (10^3^/μL)	ESR (mm/hr)	CRP (mg/L)	Anti	Dose (mg/Day)	Day ^1^	Anti	Dose (mg/Day)	Day ^2^	Anti	Conc. (mg Anti/ g Cement)
1	7.77	35.0	0.9	Uba	1200	21	AUG	1875	7	VCM	45
2	5.46	88.0	37.9	VCM	2000	12	AUG	1875	14	-	-
							Citopcin	1000			
3	7.54	62.0	11.5	Uba	2000	4	AUG	1875	14	-	-
				Tobra	75		Citopcin	1000			
4	6.81	6.0	0.5	VCM	2000	14	AUG	1875	14	-	-
							Citopcin	1000			
5	7.88	3.2	11.2	Flu	1000	2	AUG	1875	40	-	-
							Citopcin	1000			
							Fullgram	450			
6	3.91	26.0	51.4	VCM	2000	14	AUG	1875	21	-	-
							Citopcin	1000			
7	5.5	7.0	8.2	Uba	1200	12	AUG	1875	14	-	-
				AMK	1000		Citopcin	1000			
8	25.53	95.0	64.8	Uba	1200	28	Duricef	1000	30	VCM	45
9	8.45	49.0	2.0	Uba	1200	8	AUG	1875	14	-	-
				AMK	1000		Citopcin	1000			
10	9.69	29.0	36.0	VCM	2000	18	AUG	1875	14	-	-
							Citopcin	1000			
11	8.46	72.0	5.3	VCM	2000	7	-	-	-	-	-
12	11.2	71.0	51.6	VCM	2000	7	Omnicef	300	21		
13	8.38	28.0	0.8	-	-	-	Myambutol	800	180	-	-
							INAH	300			
							RIF	600			
							PZA	1500			
14	7.7	74.0	108.7	-	-	-	EMB	275	180	-	-
							INAH	75			
							RIF	600			
							PZA	400			

Values higher than the reference range are an underlined. ^1^ Number of days on which IV treatment was administered; ^2^ number of days on medication. Abbreviations: NO, number; IV, intravenous; PO, per os; anti, antibiotics; WBC, white blood cell; ESR, erythrocyte sedimentation rate; CRP, C-reactive protein; Conc, concentration; Uba, Ubacillin; VCM, vancomycin; Tobra, tobramycin; Flu, Flumarin; AMK, amikacin; AUG, Augmentin; INAH, isoniazid; RIF, rifampicin; PZA, pyrazinamide; EMB, ethambutol.

## Data Availability

The data presented in this study are available on request from the corresponding author.

## Supplementary Materials

The following supporting information can be downloaded at: https://www.mdpi.com/article/10.3390/jcm15010129/s1, Supplementary Materials S1. Summary of classifications.

## Abbreviations

C-M classification	Ciemy-Mader classification
G-A classification	Gustilo-Anderson classification
BM	bone marrow
SSI	surgical site infection
MTB	Mycobacterium tuberculosis
CoNS	coagulase-negative Stap/rylococcus
GBS	Streptococcus, beta-hemolytic, Group B
C. albicans	Candida albicans
S. marcescens	Serratia marcescens
MRSA	methicillin-resistant Staplylococcus aures
MSSA	methicillin-sensitive Staphylococcus aures
CP	cerebral palsy
SAH	subarachnoid hemorrhage
TIDM	type 1 diabetes mellitus
Uba	ubacillin
VCM	vancomycin
Tobra	tobramycin
Flu	flumarin
AMK	amikacin
AUG	Augmentin
INAH	isoniazid
RIF	rifampicin
PZA	pyrazinamide
EMB	ethambutol
